# Toxic Shock Syndrome: Eighteen Years of Experience in a Pediatric Intensive Care Unit

**DOI:** 10.7759/cureus.52898

**Published:** 2024-01-25

**Authors:** Inês Cascais, Ana Losa, Cláudia Correia, Diana H Pinto, Daniel Meireles, Alzira Sarmento

**Affiliations:** 1 Department of Pediatrics, Centro Materno-Infantil do Norte, Centro Hospitalar Universitário de Santo António, Porto, PRT; 2 Department of Pediatrics, Centro Hospitalar Entre Douro e Vouga, Santa Maria da Feira, PRT; 3 Department of Pediatric Intensive Care, Centro Materno-Infantil do Norte, Centro Hospitalar Universitário de Santo António, Porto, PRT

**Keywords:** paediatric intensive care unit (picu stay), toxic shock syndrome, staphylococcus, streptococcus, pediatrics

## Abstract

Introduction

Toxic shock syndrome (TSS) is a life-threatening disease usually caused by a *Staphylococcus aureus* or group Aβ-hemolytic* Streptococcus *infection.

Methods

In this retrospective study, we included patients with TSS admitted to a tertiary hospital’s pediatric intensive care unit (PICU) over the last 18 years. We compared the staphylococcal TSS (Staph-TSS) and streptococcal TSS (Strep-TSS) groups.

Results

We included 17 patients (64.7% male), with a median age of 6.1 years (3.0 years for streptococcal TSS versus 13.3 years for staphylococcal TSS, p = 0.040), a median of 3.0 days from symptom onset to diagnosis, and a median of 6.0 days of hospitalization. Ten patients met the Centers for Disease Control and Prevention (CDC) criteria for staphylococcal TSS (one menstrual-related) and seven met the criteria for streptococcal TSS (four of them occurring since the COVID-19 pandemic was declared). Fifteen patients had identified risk factors, primarily cutaneous lesions (29.4%). In 15 patients, at least three organs or systems were affected, with fever, rash, and hypotension as universal findings. Mucous membrane hyperemia was present in 16 patients, gastrointestinal symptoms in 14 patients, and desquamation in nine. Muscular involvement was present in seven patients, all with staphylococcal TSS (p = 0.010). All patients received two or more antibiotics, including a protein synthesis inhibitor (except for one), and required fluid resuscitation and vasoactive amines (median three days). Six patients needed invasive mechanical ventilation (median seven days). Albumin infusion was necessary in six patients, significantly more frequently in patients with streptococcal TSS (p = 0.035). Two patients with staphylococcal TSS died, while the seven patients with streptococcal TSS survived hospital discharge. There were no recurrent cases.

Conclusions

Our study revealed TSS severity and multiorgan involvement, emphasizing the importance of early diagnosis and intervention. Risk factors were prevalent, and we noted an increased frequency of group A streptococcal (GAS) TSS post-COVID-19 pandemic.

## Introduction

Toxic shock syndrome (TSS) is a severe life-threatening disease characterized by hypotension, high fever, rash, the involvement of three or more organ systems, and desquamation during convalescence [1]. It was first described in 1978 by Tood et al. [2] in a series of seven children. The first published cases were associated with infection by *Staphylococcus aureus*, and in the 1980s, cases of TSS caused by group A β-hemolytic Streptococcus were described [3]. In the early 1980s, cases of staphylococcal TSS (Staph-TSS) peaked in the United States (US) in women who used vaginal tampons with polyacrylate fibers [4], followed by a decline in menstrual TSS due to mandatory tampon labeling and improved patient education [5]. Since 1986, Staph-TSS has had a stable incidence of 0.8 to 3.4 cases per 100,000 [6] and 0.4 per 100,000 in children [5]; at least half of these cases are unrelated to menstruation [7]. The estimated incidence of group A streptococcal (GAS) bacteremia and/or invasive infection in children is one to three cases per 100,000 per year; it is even higher in infants less than one-year-old (three to five cases per 100,000 per year) [8,9]. TSS occurs in 5-15% of cases of invasive GAS infections in children [10]. Although streptococcal TSS (Strep-TSS) is less common than staph-TSS, it is associated with a significantly higher mortality rate. TSS mortality rates in children are 5-10% for strep-TSS (vs. 30-80% in adults) and 3-5% for staph-TSS [1,11]. Recent studies that included critically ill children have reported a case-fatality rate between 20% and 30% for strep-TSS cases [12-14].

TSS is defined as an acute illness secondary to an infection with toxin-producing microorganisms. These exotoxins (superantigens), mainly produced by *S. aureus*-toxic shock syndrome toxin-1 (TSST-1) and enteroxins A, B, C, D, E, and H-and group A β-hemolytic Streptococcus-pyrogenic exotoxins A, B, C, etc.; mitogenic exotoxin Z; NAD-glycohydrolase (NADase); and fragments of the M protein [15,16] - are responsible for the initiation of non-specific, polyclonal T cell activation and the consequent exaggerated immune response and cytokine storm [17]. This uncontrolled immune response is responsible for the characteristic symptoms of TSS.

The identification of TSS is based on clinical and laboratory diagnostic criteria defined by the US Centers for Disease Control and Prevention (CDC) [18,19]. This diagnosis can be challenging in the early stages of the disease, given that some of these criteria may be transient, lacking, or of late occurrence. Therefore, a high index of suspicion is needed for the diagnosis and initiation of treatment that can change the prognosis [20]. Timely resuscitation, removal of the source of infection, and tailored antimicrobial therapy with an antitoxin effect (like clindamycin, rifampicin, or linezolid) to reduce exotoxin production are essential [21,22]. In recent years, adjuvant therapy with intravenous immunoglobulin (IVIG) has been postulated to counteract the superantigens and reduce the intensity of the inflammatory cascade, effective in both streptococcal and staphylococcal TSS, particularly in severe cases [23-25]. Corticosteroids are also beneficial as coadjuvants in refractory cases, and catecholamines are resistant [26].

Studies on TSS epidemiology, clinical manifestations, diagnosis, and management have been primarily based on adult patients [1]. This study aimed to compare the characteristics and outcomes of Staph-TSS and Strep-TSS in pediatric patients.

## Materials and methods

Study design, settings, and patients

This study complied with all relevant national regulations and institutional policies and followed the tenets of the Declaration of Helsinki. The Clinical Research Department and Ethical Committee of Centro Hospitalar Universitário de Santo António and Institute of Biomedical Sciences Abel Salazar approved this study, 2023-223 (185-DEFI/177-CE).

A retrospective study was performed with a sample of pediatric patients with TSS admitted to the pediatric intensive care unit (PICU) at Centro Hospitalar Universitário de Santo António from August 2005 to August 2023. The inclusion criteria were age 0-18 years and TSS diagnosis according to the CDC criteria [18,19]. We compared the strep-TSS and staph-TSS groups.

The following data were collected from clinical records: demographic characteristics (sex, age at admission, days of hospitalization, and days until diagnosis); classification as Strep-TSS or Staph-TSS; identified risk factors for TSS; clinical findings (skin and mucous membrane manifestations, desquamation, the presence of fever and hypotension, and multiorgan involvement); laboratory data; the instituted therapy; and the results of complementary examinations, complications, and evolution during hospitalization.

Statistical analysis

Data were collected, processed, and analyzed with IBM SPSS Statistics Version 27 (IBM Corp., Armonk, NY). Continuous variables with a normal distribution are presented as the mean and standard deviation; continuous variables with a non-normal distribution are presented as the mean and interquartile (IQR) range; and categorical variables are presented as proportions (percentages). The Kolmogorov-Smirnov test was used to determine whether the variables were normally distributed. Categorical variables were analyzed with the Pearson chi-square test or Fisher’s exact test if the assumptions were not met. Continuous variables with a normal distribution were analyzed with the independent-sample t-test, while continuous variables with a non-normal distribution were analyzed with the Mann-Whitney U test. A p-value ​​<0.05 was considered to indicate a significant difference.

## Results

**Figure 1:**
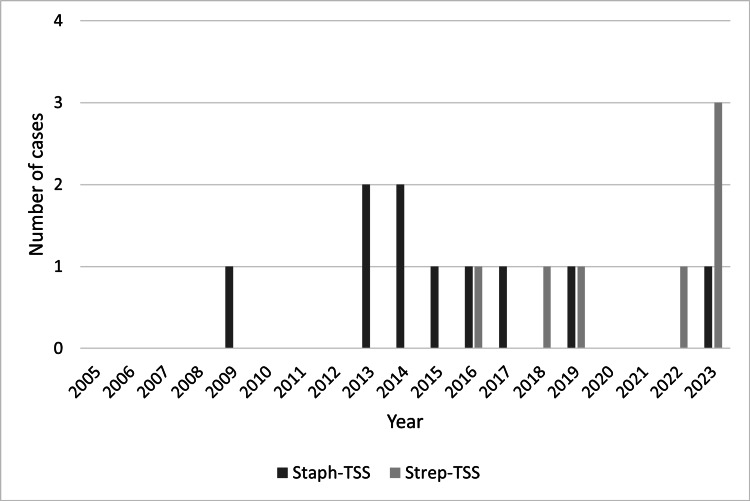
The frequency of toxic shock syndrome cases during the study period. Staph-TSS: Staphylococcal toxic shock syndrome; Strep-TSS: Streptococcal toxic shock syndrome.

TSS was diagnosed in 17 pediatric patients. Ten patients (58.8%) met the CDC case definition for Staph-TSS: six confirmed, four probable, and one related to menstruation. Seven patients (41.2%) met the criteria for Strep-TSS: four probable and three confirmed cases. Five of these seven (71.4%) Strep-TSS cases have occurred since May 2019. Figure 1 shows the annual frequency of TSS cases.

The sample demographics according to the TSS groups are depicted in Table 1. There were 11 (64.7%) males, with no significant difference between the TSS types based on sex. The median age at PICU admission was 6.1 (IQR 2.8-14.5) years. Strep-TSS cases were significantly younger (3.0 vs. 13.3 years, p = 0.040). There were a median of 3.0 (IQR 2.5-4.5) days from symptom onset to diagnosis and a median of 6.0 (IQR 4.0-9.0) days of hospitalization.

**Table 1:** Sample demographic data and risk factors for the toxic shock syndrome groups. IQR: interquartile range; PELOD: Pediatric Logistic Organ Dysfunction; PRISM: Pediatric Risk of Mortality; SD: standard deviation; Staph-TSS: staphylococcal toxic shock syndrome; Strep-TSS: streptococcal toxic shock syndrome. *Fisher's exact test; ^§^Mann-Whitney U test; ^#^independent-samples t-test.

	Staph-TSS n = 10	Strep-TSS n = 7	Total n = 17	p-value
Male, n (%)	6 (60.0)	5 (71.4)	11 (64.7)	1.000*
Median age at admission, years (IQR)	13.3 (3.8–15.1)	3.0 (2.1–8.7)	6.1 (2.8–14.5)	0.040^§^
Median time to diagnosis, days (IQR)	3.0 (3.0–3.5)	4.0 (2.0–5.0)	3.0 (2.5–4.5)	0.800^§^
Median hospitalization in intensive care days (IQR)	7.0 (3.8–8.8)	5.0 (4.0–10.0)	6.0 (4.0–9.0)	0.844^§^
Mean PRISM score (SD)	8.6 (5.9)	9.0 (4.6)	8.8 (5.2)	0.882^#^
Median PRISM probability of death, % (IQR)	2.5 (1.0–10.0)	3.0 (2.0–9.0)	3.0 (1.5–9.0)	0.459^§^
Median PELOD score (IQR)	10.5 (10.0–16.0)	10.0 (2.0–10.0)	10.0 (8.5–12.5)	0.085^§^
Median PELOD probability of death, % (IQR)	1.8 (1.0–14.8)	1.0 (1.0–1.7)	1.3 (1.0–3.1)	0.295^§^
Deaths, n (%)	2 (20.0)	0	2 (11.8)	0.485*
Risk factors				
Cutaneous lesions, n (%)	2 (20.0)	3 (42.9)	5 (29.4)	0.593*
Recent surgery, n (%)	2 (20.0)	1 (14.3)	3 (17.6)	1.000*
Varicella infection, n (%)	2 (20.0)	1 (14.3)	3 (17.6)	1.000*
Respiratory tract infections, n (%)	2 (20.0)	1 (14.3)	3 (17.6)	1.000*
Tampon usage, n (%)	1 (10.0)	0	1 (5.9)	1.000*

On admission, the mean Pediatric Risk of Mortality (PRISM) score was 8.8 (median probability of death of 3.0%), and the median Pediatric Logistic Organ Dysfunction (PELOD) score was 10.0 (median probability of death of 1.3%), with no significant difference between the TSS types. Two (11.8%) patients with Staph-TSS died. There were no recurrent cases. None of the patients were immunocompromised. The risk factors for TSS by group are described in Table 1. They were identified in 15 patients (88.2%), with cutaneous lesions (29.4%) being the most common.

Health outcomes, multiorgan involvement, and treatment

As a multisystemic disease, TSS has a broad spectrum of signs and symptoms that vary in severity. Table 2 shows the clinical manifestations according to the TSS groups. In 15 patients (88.2%), at least three organs or systems were affected, with fever, rash, and hypotension as universal findings. Mucous membrane hyperemia occurred in 16 cases (94.1%), most commonly in the oropharynx, and was present in all seven patients with Strep-TSS. Desquamation occurred during hospitalization in nine patients (52.9%); two-thirds of these patients had Staph-TSS. Gastrointestinal symptoms, renal impairment, and liver and hematological involvement were more frequent in the Staph-TSS group. Muscular involvement was present in seven cases, all with Staph-TSS, with a significant difference between the TSS groups (p = 0.010). Central nervous system (CNS) involvement and acute respiratory distress syndrome were more frequent in the strep-TSS group.

**Table 2:** Clinical manifestations of the toxic shock syndrome groups. The Centers for Disease Control and Prevention criteria were used to define the involvement of each system. Staph-TSS: staphylococcal toxic shock syndrome; Strep-TSS: streptococcal toxic shock syndrome. *Fisher's exact test; N/A, not applicable.

	Staph-TSS n = 10	Strep-TSS n = 7	Total n = 17	p-value
Fever, n (%)	10 (100.0)	7 (100.0)	17 (100.0)	N/A
Rash: diffuse macular erythroderma, n (%)	10 (100.0)	7 (100.0)	17 (100.0)	N/A
With petechiae	4 (40.0)	2 (28.6)	6 (35.3)	1.000*
With vesicles or blisters	2 (20.0)	2 (28.6)	4 (23.5)	1.000*
Hypotension, n (%)	10 (100.0)	7 (100.0)	17 (100.0)	N/A
Mucous membrane hyperemia, n (%)	9 (90.0)	7 (100.0)	16 (94.1)	1.000*
Oropharyngeal, n (%)	6 (60.0)	7 (100.0)	13 (76.5)	0.103*
Conjunctival, n (%)	4 (40.0)	3 (42.9)	7 (41.2)	1.000*
Oropharyngeal and conjunctival, n (%)	1 (10.0)	3 (42.9)	4 (23.5)	0.250*
Desquamation during hospitalization, n (%)	6 (60.0)	3 (42.9)	9 (52.9)	0.637*
Gastrointestinal symptoms, n (%)	9 (90.0)	5 (71.4)	14 (82.4)	0.537*
Liver involvement, n (%)	6 (60.0)	3 (42.9)	9 (52.9)	0.637*
Renal impairment, n (%)	8 (80.0)	4 (57.1)	12 (70.6)	0.593*
Muscular involvement, n (%)	7 (70.0)	0	7 (41.2)	0.010*
Central nervous system involvement, n (%)	4 (40.0)	4 (57.1)	8 (47.1)	0.637*
Hematologic involvement, n (%)	7 (70.0)	4 (57.1)	11 (64.7)	0.644*
Acute respiratory distress syndrome, n (%)	1 (10.0)	2 (28.6)	3 (17.6)	0.537*

Laboratory evaluation (Table 3) revealed that all patients had total leukocyte counts and C-reactive protein (CRP) levels above the upper limit of normal by age, as defined by the laboratory of our hospital center. Significant elevations of leukocytes (≥ 20,000/µL) and CRP (≥ 20 mg/dL) occurred at a considerable frequency in our sample (58.8% and 70.6%, respectively), with no significant difference between the TSS groups. Procalcitonin levels have only been available in our center since 2016; thus, they have only been determined in seven cases, all with a value of ≥0.5 ng/mL. The mean leukocyte count, CRP level, procalcitonin level, and median serum lactate level were higher in the Strep-TSS group compared with the Staph-TSS group (although the differences were not significant). The mean albumin level was slightly lower in the Strep-TSS group, with hypoalbuminemia (<3 g/dL) in 71.4% of the patients.

Regarding the microbiological results, methicillin-susceptible *S. aureus* (MSSA) was isolated in six cases (four from a non-sterile site and two from a normally sterile site), and group A Streptococcus was isolated in seven cases (three from a normally sterile site and four from a non-sterile site). Bacteremia was present in only two cases with strep-TSS (p = 0.154). Concerning the antibiogram, almost all the isolated bacteria were susceptible to oxacillin, penicillin, and clindamycin, except for one case in which group A Streptococcus identified in the pleural fluid was resistant to clindamycin.

**Table 3:** Laboratory results for the toxic shock syndrome groups. CRP: C-reactive protein; IQR: interquartile range; SD: standard deviation; Staph-TSS: staphylococcal toxic shock syndrome; Strep-TSS: streptococcal toxic shock syndrome. *Fisher's exact test; ^§^Mann-Whitney U test; ^#^independent-samples t-test; N/A, not applicable.

	Staph-TSS n = 10	Strep-TSS n = 7	Total n = 17	p-value
Leukocytosis: ≥20,000/µL, n (%)	6 (60.0)	4 (57.1)	10 (58.8)	1.000*
Mean leukocyte count (SD)	20962.0 (8320.9)	23408.6 (6456.5)	21969.4 (7491.2)	0.525^#^
CRP elevation: ≥0.5 mg/dL, n (%)	10 (100.0)	7 (100.0)	17 (100.0)	N/A
CRP ≥20 mg/dL, n (%)	7 (70.0)	5 (71.4)	12 (70.6)	1.000*
Mean CRP level (SD)	22.1 (9.4)	29.7 (12.0)	25.2 (10.9)	0.163^#^
Procalcitonin elevation: ≥0.5 ng/mL, n (%)	2 (20.0)	5 (71.4)	7 (41.2)	0.058*
Mean procalcitonin level (SD)	20.6 (27.7)	49.1 (38.1)	41.0 (35.9)	0.390^#^
Severe metabolic acidosis, n (%)	5 (50.0)	1 (14.3)	6 (35.3)	0.304*
Median serum lactate level, mmol/L (IQR)	1.1 (0.8–2.9)	2.8 (1.1–6.7)	1.4 (0.8–3.6)	0.155^§^
Hypoalbuminemia: <3 g/dL, n (%)	6 (60.0)	5 (71.4)	11 (64.7)	1.000*
Mean albumin level (SD)	2.6 (0.4)	2.5 (0.6)	2.6 (0.5)	0.678^#^

As shown in Table 4, all patients received a combination of at least two antibiotics: cloxacillin plus clindamycin in six cases, vancomycin plus ceftriaxone plus clindamycin in five cases, penicillin plus clindamycin in four cases, and vancomycin plus clindamycin in one case. The patient who did not receive a protein synthesis inhibitor was treated with vancomycin and ceftriaxone for Staph-TSS, presented with refractory hypotension, and progressed to multiorgan failure with multiple massive cerebral intraparenchymal hemorrhages. The patient was pronounced brain dead and died on the eighth day of hospitalization.

All patients required fluid resuscitation and hemodynamic support with vasoactive amines (with a median duration of three days), and six patients needed invasive mechanical ventilation (with a median duration of seven days). Treatment with fresh frozen plasma, platelet transfusion, sodium bicarbonate, and albumin infusion occurred in 35.3% of the cases; the latter was significantly more frequent in the Strep-TSS group (p = 0.035). IVIG was administered to four patients, and two patients received corticosteroids. One of the three patients who required pleural effusion drainage with chest tubes died of cardiorespiratory arrest in the context of refractory hypotension, metabolic acidosis, and respiratory failure with bilateral hemothorax following thoracic and upper right arm lymphangioma surgery.

**Table 4:** Treatment for the toxic shock syndrome groups. IQR: interquartile range; SD: standard deviation; Staph-TSS: staphylococcal toxic shock syndrome; Strep-TSS: streptococcal toxic shock syndrome. *Fisher's exact test; ^§^Mann-Whitney U test; N/A, not applicable.

	Staph-TSS n = 10	Strep-TSS n = 7	Total n = 17	p-value
Fluid resuscitation, n (%)	10 (100.0)	7 (100.0)	17 (100.0)	N/A
Antibiotics: combination, n (%)	10 (100.0)	7 (100.0)	17 (100.0)	N/A
Protein synthesis inhibitor, n (%)	9 (90.0)	7 (100.0)	16 (94.1)	1.000*
Vasoactive amines, n (%)	10 (100.0)	7 (100.0)	17 (100.0)	N/A
Median duration, days (IQR)	3.5 (1.8–5.3)	3.0 (2.0–5.0)	3.0 (2.0–5.0)	0.882^§^
Invasive mechanical ventilation, n (%)	5 (50.0)	1 (14.3)	6 (35.3)	0.304*
Median duration, days (IQR)	6 (5.0–9.5)	22 (22.0–22.0)	7 (5.0–13.8)	0.137^§^
Fresh frozen plasma, n (%)	4 (40.0)	2 (28.6)	6 (35.3)	1.000*
Platelet transfusion, n (%)	3 (30.0)	3 (42.9)	6 (35.3)	0.644*
Red cell concentrate transfusion, n (%)	2 (20.0)	1 (14.3)	3 (17.6)	1.000*
Sodium bicarbonate, n (%)	5 (50.0)	1 (14.3)	6 (35.3)	0.304*
Albumin infusion, n (%)	1 (10.0)	5 (71.4)	6 (35.3)	0.035*
Intravenous immunoglobulin, n (%)	1 (10.0)	3 (42.9)	4 (23.5)	0.250*
Corticosteroids, n (%)	1 (10.0)	1 (14.3)	2 (11.8)	1.000*
Chest tube, n (%)	2 (20.0)	1 (14.3)	3 (17.6)	1.000*

## Discussion

This retrospective study has revealed the characteristics of TSS in a pediatric sample admitted to a PICU over almost two decades. We have reported the distribution of cases over the years, the broad spectrum of severity and multiorgan involvement of TSS, and the differences between the Staph-TSS and Strep-TSS groups.

After the coronavirus disease 2019 (COVID-19) pandemic, several European countries reported an increase in invasive streptococcal disease, particularly among children under 10 years of age, in the autumn and winter of 2022 compared with previous years [27]. This increase could be attributed to the onset of the GAS infection season occurring ahead of schedule, coinciding with the increased circulation of respiratory viruses and the co-occurrence of viral infections, which may increase the likelihood of invasive GAS disease [27,28]. The post-COVID-19 pandemic increase in strep-TSS cases, which we verified mainly since 2022, is similar to a recent report [29]. However, it is not definitively established whether this increase reflects an actual shift in epidemiology or alterations in testing practices and characteristics. Further studies are needed to clarify the underlying factors driving these observed trends.

The sex prevalence of TSS in this study was slightly higher for males (64.7%), unlike similar studies (44.4% in a 15-year study at a Spanish PICU [30] and 36.7% in a six-year study at a French PICU [31] and a one-year study at United Kingdom pediatric and burn units), but closer to the 51.6% in an 11-year study at two Australian tertiary pediatric referral centers [32]. The fact that our sample tended to be younger (median age of six years) may have contributed to this result, considering that there will be a lower probability of recent tampon use, a preponderant risk factor for staph-TSS. Nevertheless, there were no significant differences based on sex between the TSS types.

Cutaneous lesions were the most common risk factor, with strep-TSS cases showing the highest frequency. This finding aligns with the fact that approximately 30-40% of GAS infections are linked to recent skin injuries, often minor trauma [10]. Moreover, GAS bacteremia typically occurs secondary to a primary site of infection, most commonly in the skin and soft tissues [33], with a greater incidence in younger children, particularly those under one year old [8].

Similar to Javouhey et al. [31], patients with strep-TSS were significantly younger at PICU admission, had higher leukocyte counts and CRP levels, and more frequently had acute respiratory distress syndrome. Moreover, the strep-TSS group had a higher frequency of bacteremia, in agreement with other studies [1,34]. Desquamation; gastrointestinal symptoms; and renal, liver, and hematologic (mostly thrombocytopenia) impairment were also more frequent in patients with Staph-TSS in the study by Javouhey et al. [31], although the difference was not statistically significant. In contrast, our Strep-TSS cases did not present with significantly higher organ dysfunction scores or longer PICU stays. In similar studies, Strep-TSS cases required invasive ventilation more frequently [31,32] and for a longer time [31] than Staph-TSS cases. In our study, half of the patients with Staph-TSS required ventilatory support, while only one patient with Strep-TSS required this intervention. Thus, we could not draw comparative conclusions regarding the duration of ventilatory support. However, the patient with Strep-TSS was intubated for a total of 22 days, longer than the median duration for the Staph-TSS cases (six days).

Consistent with the CDC case definition criteria, hypotension was present in all of our patients. All patients required amine support, which was higher than that reported by Javouhey et al. [31] (73% considering all patients, 67% for staph-TSS, and 80% for Strep-TSS), Costa Orvay et al. [30] (56% considering all patients, 100% for Staph-TSS, and 43% for Strep-TSS), Chen et al. [32] (71% considering all patients), and Adalat et al. [14] (67% considering all patients). However, the last two studies included patients who were not admitted to the PICU, which may explain the lower percentage reported. The duration of treatment with amines (three days) was similar to that reported by Costa Orvay et al. [30] and Adalat et al. [14]. Fever and rash were also universal symptoms in our study, similarly to Costa Orvay et al. [30] The rash frequency was close to that reported by Adalat et al. [14] (96%) and was more frequent in streptococcal cases in our patients than reported by Javouhey et al. [31] (73%). Mucous membrane hyperemia was slightly more frequent in the strep-TSS versus staph-TSS group than in the study by Adalat et al. [14].

In TSS cases, interleukin 1 produced by activated T cells mediates skeletal muscle proteolysis and likely contributes to myalgia and weakness complaints and elevated creatine phosphokinase (CPK) levels [35]. Muscular manifestations (defined as severe myalgia or a CPK level at least twice the upper limit of normal) are one of the multisystem involvement CDC criteria for staph-TSS [19], and severe myalgias are more frequent in this group [1]. We identified seven cases of muscular involvement, all staph-TSS, with a significant difference compared with the strep-TSS group.

The higher frequency of CNS involvement in Strep-TSS cases can be explained by the fact that GAS-associated sepsis syndrome can induce CNS inflammation, either directly by bacteria penetrating the brain and mediated by autoantibodies recognizing CNS epitopes or via secondary subcutaneous infections and systemic inflammation [36].

The usual laboratory findings in Staph-TSS include increased immature neutrophils, thrombocytopenia, and anemia; disseminated intravascular coagulation may also be present [1]. Thus, the higher frequency of treatment with fresh frozen plasma and red cell concentrate transfusions in this group is understandable. Thrombocytopenia is common in both Staph-TSS and Strep-TSS [34], which is reflected by the fact that in the six cases that required platelet transfusion, there were three Staph-TSS cases and three Strep-TSS cases.

Differently from what has been described in the literature [1,11,14,32], although Strep-TSS was less frequent in our sample, the number of deaths was not higher in this group. Indeed, the two patients who died in the present study had Staph-TSS. Despite the absence of a post-mortem study, our hypothesis regarding the death cases is that they were probably related to the host-pathogen interaction and disease severity. However, this might also be attributed to the small sample size, which hinders us from establishing definitive conclusions.

Albumin is an antioxidant and a primary extracellular scavenger that provides amino acids that are essential for cellular and matrix synthesis. Inflammatory conditions lead to hypoalbuminemia through heightened capillary permeability, resulting in serum albumin leakage and a reduced half-life [37]. Hypoalbuminemia is an established independent risk factor and a mortality indicator in critically ill patients [38]. It is also a common laboratory finding in patients with Strep-TSS [39]. Therefore, its presence in the two patients who died and the significantly higher frequency of albumin infusions needed in the Strep-TSS cases are understandable.

Empiric treatment for TSS consists of flucloxacillin for *Streptococcus pyogenes* and *S. aureus* or vancomycin for suspected methicillin-resistant *S. aureus* [MRSA] or hospital-acquired infections. Ceftriaxone is added for possible Gram-negative infections and piperacillin/tazobactam for suspected Pseudomonas, other Gram-negative bacteria, or in specific cases like post-abortion or gynecological procedures. Clindamycin is also commonly added to the regimen due to its effectiveness in inhibiting protein synthesis and suppressing the production of bacterial toxins. Indeed, multiple studies have demonstrated improved TSS survival when adding clindamycin to antibiotic regimens [22,23,31]. The recommended antimicrobial treatment for staph-TSS is flucloxacillin (if caused by MSSA) or cefazolin or vancomycin (if caused by MRSA), plus clindamycin or linezolid (if *S. aureus* is resistant to clindamycin or vancomycin or there is a β-lactam allergy or there is vancomycin resistance). For strep-TSS, the recommended treatment is penicillin G or cefazolin (if there is hypersensitivity to β-lactams without anaphylaxis) or vancomycin (if there is anaphylaxis to β-lactams), plus clindamycin or linezolid (if *S. pyogenes* is resistant to clindamycin). Evidence supports the efficacy of IVIG and its role as an adjuvant treatment option in managing TSS because it neutralizes the superantigen and halts cytokine production in Staph-TSS and Strep-TSS [25]. A systematic review of Strep-TSS demonstrated that clindamycin associated with IVIG reduces mortality from 33.7% to 15.7% [24]. In our small sample, we found a higher mortality rate for staph-TSS than what has typically been reported for children [1,11]. Of the two deaths with Staph-TSS, one received no antitoxin therapy (clindamycin or IVIG), and the other was recently submitted to thoracic and upper right arm lymphangioma surgery that was complicated with respiratory failure with bilateral hemothorax, which may have contributed to the death.

Strengths and limitations

The major strength of this study was the 18 years of data collection, which allowed for a comprehensive characterization of pediatric patients with TSS admitted to the PICU at a tertiary pediatric hospital and enhanced the internal validity of the findings.

However, this study has some limitations. First, like any retrospective study, it has inherent limitations associated with data collection from medical records, including missing or incomplete information exacerbated by the prolonged period selected for data collection. Furthermore, our sample of patients admitted to a single tertiary pediatric hospital may not fully represent the general population of pediatric patients with TSS. Referral bias may have influenced the findings, and the results may not be generalizable to other healthcare settings or regions. The study design restricts the ability to establish causal relationships or to assess longitudinal changes over time or long-term outcomes. Finally, the lack of a control group limits the ability to make direct comparisons or draw definitive conclusions regarding the observed associations. Despite these limitations, this study provides valuable insights into the clinical characteristics and associated conditions of pediatric patients with TSS, facilitating better understanding and management of this complex disorder.

## Conclusions

In conclusion, our study provides a thorough understanding of the spectrum of severity and multiorgan involvement in pediatric TSS. The diverse clinical conditions observed highlight the need for a pediatric-specific standard of care for the intensive management of these patients to improve their prognosis and health outcomes. The standard for pediatric TSS has evolved throughout these 18 years. Furthermore, the combination of antibiotic therapy varies among hospital centers, as different pathogen susceptibilities and drug availability may interfere with local protocols. In addition, the epidemiology of TSS appears to be changing, and our findings align with the most recent published literature.

Additionally, this research emphasizes the importance of early diagnosis, regular surveillance, appropriate intervention, and support across multiple domains in the pediatric population. This study presented an opportunity to review and adjust protocols within our unit, according to our results and the most recent scientific evidence, and should contribute to the update of evidence-based guidelines for the care of these pediatric patients. Multicentric studies using validated outcomes are needed to provide comprehensive data on the epidemiology, prevalence, and severity of conditions associated with this complex rare disease, document their long-term impact on quality of life, and investigate treatment and management options.
